# Efficacy and safety of enavogliflozin vs. dapagliflozin as add-on therapy in patients with type 2 diabetes mellitus based on renal function: a pooled analysis of two randomized controlled trials

**DOI:** 10.1186/s12933-024-02155-9

**Published:** 2024-02-15

**Authors:** Young Sang Lyu, Sangmo Hong, Si Eun Lee, Bo Young Cho, Cheol-Young Park

**Affiliations:** 1 Division of Endocrinology and Metabolism, Department of Internal Medicine, Chosun University Hospital, Gwangju, Republic of Korea; 2 Division of Endocrinology, Department of Internal Medicine, Hanyang University Guri Hospital, 153 Gyeongchun-ro, Guri, 11923 Korea; 3 Daewoong Pharmaceutical Co., Ltd, Seoul, Republic of Korea; 4 Division of Endocrinology and Metabolism, Department of Internal Medicine, Kangbuk Samsung Hospital, Sungkyunkwan University School of Medicine, 29 Saemunan-ro, Jongno-gu, Seoul, 03181 Korea

**Keywords:** Enavogliflozin, Glycemic control, Non-insulin dependent diabetes mellitus, SGLT-2 inhibitor, Urine glucose excretion rate

## Abstract

**Background:**

We assessed the efficacy and safety of enavogliflozin (0.3 mg), a newly developed SGLT-2 inhibitor, in patients with type 2 diabetes mellitus based on kidney function via pooled analysis of two 24-week, randomized, double-blind phase III trials.

**Methods:**

Data from 470 patients were included (enavogliflozin: 0.3 mg/day, *n* = 235; dapagliflozin: 10 mg/day, *n* = 235). The subjects were classified by mildly reduced (60 ≤ eGFR < 90 mL/min/1.73 m², *n* = 247) or normal eGFR (≥ 90 mL/min/1.73 m², *n* = 223).

**Results:**

In the mildly reduced eGFR group, enavogliflozin significantly reduced the adjusted mean change of HbA1c and fasting plasma glucose levels at week 24 compared to dapagliflozin (− 0.94% vs. −0.77%, *P* = 0.0196). Enavogliflozin exhibited a more pronounced glucose-lowering effect by HbA1c when combined with dipeptidyl peptidase-4 inhibitors than that observed in their absence. Enavogliflozin showed potent blood glucose-lowering effects regardless of renal function. Conversely, dapagliflozin showed a significant decrease in the glucose-lowering efficacy as the renal function decreased. Enavogliflozin showed a higher urinary glucose excretion rate in both groups. The homeostatic model assessment showed that enavogliflozin markedly decreased the insulin resistance. The blood pressure, weight loss, or homeostasis model assessment of beta-cell function values did not differ significantly between enavogliflozin and dapagliflozin. Adverse events were similar between both drugs.

**Conclusions:**

The glucose-lowering efficacy of enavogliflozin is superior to that of dapagliflozin in patients with type 2 diabetes mellitus with mild renal function impairment; this is attributed to its potent urinary glucose excretion-promoting ability. The emergence of new and potent SGLT-2 inhibitors is considered an attractive option for patients with inadequate glycemic control and decreased renal function.

**Trial registration:**

Not applicable (pooled analysis).

**Supplementary Information:**

The online version contains supplementary material available at 10.1186/s12933-024-02155-9.

## Background

Sodium-glucose cotransporter-2 (SGLT-2) inhibitors are a class of antidiabetic therapies that inhibit the sodium-glucose cotransporter-2 protein located in the kidney proximal tubules. By blocking glucose reabsorption, these drugs promote urinary glucose excretion (UGE) and improved glycemic control [1]. As implied by the SGLT-2 mechanism, the glucose-lowering efficacy of SGLT-2 inhibitors slows the kidney function decline. Even in patients with stage 2 chronic kidney disease, with an estimated glomerular filtration rate (eGFR) of ≥ 60 to < 90 mL/min/1.73 m², the glucose-lowering efficacy of SGLT-2 inhibitor is lower than that in those with a normal kidney function, with an eGFR of ≥ 90 mL/min/1.73 m² [2].

Enavogliflozin is a newly developed potent SGLT-2 inhibitor [3–6]. In a study comparing enavogliflozin 0.3 mg and dapagliflozin 10 mg in healthy adults, the post-treatment UGE was higher with the former treatment than with the latter. In a pharmacokinetic and pharmacodynamic study, the potent effect of enavogliflozin could be explained by its selective and competitive inhibition of SGLT-2 compared to that of other SGLT-2 inhibitors and its higher distribution in the kidneys [4, 7]. The strong affinity of enavogliflozin for the kidneys and its prolonged inhibitory effect on SGLT-2 may further contribute to its notable glucose-lowering efficacy.

Recently, two randomized controlled phase III studies investigated the efficacy and safety of enavogliflozin 0.3 mg and compared them with those of dapagliflozin 10 mg in patients with inadequate blood glucose control, using metformin monotherapy [8] or a combination of metformin and the dipeptidyl peptidase-4 inhibitor (DPP-4i) gemigliptin [9]. In these studies, enavogliflozin exhibited comparable glucose-lowering efficacy to dapagliflozin. An interesting observation is that enavogliflozin significantly increased the post-UGE to a greater extent than dapagliflozin. Furthermore, in the subgroup analysis of both studies, the blood glucose-lowering efficacy of enavogliflozin was more pronounced in patients with mildly reduced kidney function, having an eGFR of ≥ 60 to < 90 mL/min/1.73 m². But, the relatively small number of participants in each study was not enough to produce statistically significant results in comparing the blood glucose-lowering effects between the two SGLT-2 inhibitors, emphasizing the necessity for studies with a larger number of participants.

Due to the eGFR-dependent glucose-lowering effect of SGLT-2 inhibitors, a medication with superior post-treatment UGE may be more advantageous in patients with impaired renal function. Although several studies have compared the glucose-lowering effects of SGLT-2 inhibitors, few studies have compared the efficacy of these medications based on renal function. So, we aimed to evaluate the efficacy and safety of enavogliflozin compared with those of dapagliflozin through a pooled analysis stratified by renal function.

## Methods

### Study design

We analyzed data from two randomized, double-blind, placebo-controlled, phase III trials comparing enavogliflozin 0.3 mg with dapagliflozin 10 mg (Table 1). In both studies, the patients underwent a stabilization period and an initial run-in. The patients were then randomized to receive daily doses of enavogliflozin 0.3 mg or dapagliflozin 10 mg for 24 weeks. In one phase III study, the patients with inadequate glycemic control, despite receiving metformin monotherapy (≥ 1,000 mg/day), were candidates for screening [8]. In the other phase III study, patients with inadequate glycemic control, despite receiving a combination of metformin (≥ 1,000 mg/day) and gemigliptin therapies, were screened [9]. In these two studies, 274 and 385 patients were expected to be enrolled, respectively; however, 200 and 270 patients were ultimately included and randomized, respectively.

**Table 1:** Randomized, double-blind, active-controlled Phase III trials included in this pooled analysis

Clinical Trials.gov registration number	Background treatment	Treatment arms	Mean age (years)	Men (%)	Treatment period (weeks)	Reference
NCT04634500	Metformin alone	Enavogliflozin 0.3 mg	59.03	58.42	24	KA Han. et al. [8]
		Dapagliflozin 10 mg	60.35	54.55		
NCT04654390	Combination of metformin and gemigliptin	Enavogliflozin 0.3 mg	58.14	54.48	24	KS Kim, et al. [9]
		Dapagliflozin 10 mg	59.07	50.00		

Throughout the study, the metformin treatment remained unchanged, with no modifications in dosage, administration frequency, or type of metformin product (immediate or extended release). The use of other antidiabetic medication, glucose or glucagon injections, weight loss medications, contrast agents containing iodine, or medications with potential interactions with the study drugs or metformin was not allowed during the study. Follow-up appointments were arranged every six weeks (at 6, 12, 18, and 24 weeks) after the randomization process for efficacy and safety evaluations.

### Patients

The inclusion criteria were almost consistent in both phase III studies. Participants had to be between 19 and 80 years old and have a body mass index (BMI) of between 20 and 45 kg/m². In the metformin combination study, individuals with HbA1c values of 7.0% (53 mmol/mol) to 10.5% (91 mmol/mol) and FPG levels < 270 mg/dL during the screening process were eligible for inclusion. In another metformin/gemigliptin combination study, individuals with HbA1c values of 7.0% (53 mmol/mol) to 11% (97 mmol/mol) and FPG levels < 270 mg/dL during the screening process were included. The exclusion criteria of both phase III studies were as follows: systolic blood pressure (SBP) > 180 mmHg or diastolic blood pressure (DBP) > 110 mmHg, heart failure, an eGFR level below 60 mL/min/1.73 m², aspartate aminotransferase or alanine aminotransferase levels exceeding 3 times the upper limit of the normal range, and any other conditions or diseases of clinical significance.

### Efficacy and safety assessments

Conventional efficacy and safety monitoring protocols were applied throughout the entire treatment period in both phase III trials. The patients visited the study site for evaluation of both efficacy and safety at weeks 6, 12, 18, and 24.

In this pooled analysis, the patient data were analyzed based on kidney function. Kidney function is assessed by the eGFR according to the modification of diet in the renal disease study equation. A normal eGFR is defined as ≥ 90 mL/min/1.73 m² and a mildly reduced eGFR is defined as ≥ 60 to < 90 mL/min/1.73 m². The efficacy endpoints included the changes from baseline to week 24 in HbA1c, FPG, body weight, urine glucose-to-creatinine ratio (UGCR), homeostasis model assessment of β-cell function (HOMA- β), homeostasis model assessment of insulin resistance (HOMA-IR), and blood pressure. Glycemic responses were also assessed using the following criteria: HbA1c level < 7.0% (53 mmol/mol); HbA1c level < 6.5% (48 mmol/mol); HbA1c level < 7.0% (53 mmol/mol) or HbA1c level reduction > 0.5%; HbA1c level < 7.0% (53 mmol/mol) or HbA1c level reduction > 0.7%; HbA1c level < 7.0% (53 mmol/mol) or HbA1c level reduction > 1.0%.

Safety was evaluated by collecting the treatment-emergent adverse events (TEAEs), with a special focus on monitoring hypoglycemia, vaginal infection, urinary tract infection, genital infection, and pollakiuria occurrences.

### Statistical analysis

The pooled clinically relevant data from the individual trials were compared between the enavogliflozin and dapagliflozin groups at baseline and at weeks 6, 12, 18, and 24. The data were derived from the randomized group and presented as the number of patients (percentage) for categorical variables and the mean (standard deviation) for the continuous variables. To assess the efficacy endpoints measured as continuous variables, group comparisons were conducted using an analysis of covariance (ANCOVA). This analysis controlled the baseline values and included the randomization stratification factors (regimen of antidiabetic drug within the last 24 weeks prior to informed consent [monotherapy or combination] (before wash-out of other antidiabetic drug) and HbA1c level [< 8% or ≥ 8%] at Visit 1 (screening) by the central laboratory) as covariates and the treatment group as treatment effect (enavogliflozin vs. dapagliflozin). Changes from the baseline to these endpoints were presented, including adjusted mean changes and differences between groups, which were calculated as the least square mean differences (enavogliflozin group – dapagliflozin group) using ANCOVA. To ensure the reliability of the HbA1c and FPG level results, a subgroup analysis was performed.

The univariate association between eGFR and UGCR was examined using Pearson’s correlation coefficient. Correlations were conducted separately for enavogliflozin 0.3 mg, dapagliflozin 10 mg, and the combined group of both medications. Additionally, plots were used to visually demonstrate the differences in UGCR between the two medications.

For the efficacy analysis, the per-protocol set was the primary analysis set, and all safety assessments were conducted on the safety set. All statistical analyses were performed using a two-sided approach and a significance level of 5%. Statistical analyses were conducted using Statistical Analysis Software (SAS) version 9.4 (SAS Institute Inc., Cary, North Carolina, USA). Detailed descriptions of the adverse events were coded according to the Medical Dictionary for Regulatory Activities (MedDRA) version 24.0.

## Results

### Disposition of patients and baseline characteristics

Baseline characteristics of the pooled analysis are presented in Table 2. The pooled analysis included 470 randomized patients with type 2 diabetes mellitus, of whom 235 received enavogliflozin and 235 received dapagliflozin. When classified based on renal function, the normal eGFR group (eGFR ≥ 90 mL/min/1.73 m²) consisted of 247 patients, while the mild reduced eGFR group (eGFR ≥ 60 to < 90 mL/min/1.73 m²) included 223 patients. In the normal eGFR group, the average age was approximately 56 years for both the enavogliflozin and dapagliflozin groups, and the mean time since the diagnosis of type 2 diabetes mellitus was 8.39 and 7.48 years, respectively. The average BMI in both groups was 26 kg/m^2^; the HbA1c levels of the two groups were comparable (7.9% [63 mmol/mol]), with no significant differences. The SBP, DBP, and weight levels did not significantly differ between the two groups.

**Table 2:** Baseline characteristics of the pooled patients

	Normal eGFR (eGFR ≥ 90 mL/min/1.73m^2^)		Mildly reduced eGFR (60 ≤ eGFR < 90 mL/min/1.73m^2^)	
	Enavogliflozin 0.3 mg (*n* = 106)	Dapagliflozin 10 mg (*n* = 117)	Enavogliflozin 0.3 mg (*n* = 129)	Dapagliflozin 10 mg (*n* = 118)
Male, n (%)	49 (46.23)	60 (51.28)	83 (64.34)	62 (52.54)
Age, years	56.13 (11.23)	56.83 (10.79)	60.49 (9.72)	62.36 (8.61)
Weight, kg	69.52 (11.68)	69.70 (12.60)	70.95 (11.65) ^a^	67.82 (10.65) ^a^
BMI, kg/m^2^	26.29 (3.88)	26.26 (3.34)	26.20 (3.22)	25.48 (3.05)
Duration of diabetes, years	8.39 (6.27)	7.48 (5.15)	10.66 (6.81)	10.38 (5.54)
OHA history, n (%)				
Metformin alone	21 (19.81)	34 (29.06)	38 (29.46)	26 (22.03)
Add-on therapy to metformin	85 (80.19)	83 (70.94)	91 (70.54)	92 (77.97)
HbA1c, %	7.93 (0.82)	7.90 (0.81)	7.90 (0.79)	7.94 (0.78)
HbA1c, mmol/mol	63.16 (8.99)	62.88 (8.81)	62.87 (8.64)	63.26 (8.55)
HbA1c ≥ 8% (64 mmol/mol), n (%)	38 (35.85)	38 (32.48)	43 (33.33)	44 (37.29)
FPG, mg/dL	165.96 (35.87)	161.27 (32.22)	160.40 (30.52)	159.71 (32.34)
eGFR, mL/min/1.73m^2^	105.18 (13.23)	106.80 (13.47)	76.71 (8.06)	77.97 (7.73)
SBP, mmHg^†^	126.67 (10.88)	125.82 (11.92)	129.37 (14.37) ^b^	125.68 (13.57) ^b^
DBP, mmHg^†^	76.49 (8.81)	75.61 (8.72)	76.77 (10.05) ^c^	74.11 (10.57) ^c^

Data are based on the randomized set and presented as number of patients (percentage) for categorical variables and mean (standard deviation) for continuous variables. Presented data are based on the data collected at the screening visit (-4 weeks from randomisation) except those marked with dagger sign (^†^), which are based on the data collected on randomisation day (Day 0). Testing for difference between Enavogliflozin and Dapagliflozin (chi-square test or Fisher’s exact test for categorical variables and two sample t-test or Wilcoxon rank sum test for continuous variables). Statistically significant between-group difference: ^a^*p*=0.0390, ^b^*p*=0.0401, ^c^*p*=0.0445, ^d^*p*=0.0021, ^e^*p*=0.0164

BMI: body mass index; DBP: diastolic blood pressure; eGFR: estimated glomerular filtration rate; FPG: fasting plasma glucose; HbA1c: glycated hemoglobin; HDL-C: high-density lipoprotein cholesterol; LDL-C: low-density lipoprotein cholesterol; OHA: oral antihyperglycemic agent; SBP: systolic blood pressure; TG: triglyceride

In the mildly reduced eGFR group, the age of patients was approximately 60.5 and 62.4 years in the enavogliflozin and dapagliflozin groups, respectively. These patients were older than the patients in the normal eGFR group and the mean time since the type 2 diabetes mellitus diagnosis was 10.66 and 10.38 years for the enavogliflozin and dapagliflozin groups, respectively, which was longer than that in the normal eGFR group. The average BMI was approximately 26.2 kg/m^2^ and 25.5 kg/m^2^ for the enavogliflozin and dapagliflozin groups, respectively. The HbA1c levels were similar between the two groups (7.9% [63 mmol/mol]). The SBP and DBP and weight levels were higher in the enavogliflozin group than in the dapagliflozin group.

### Efficacy

**Fig. 1:**
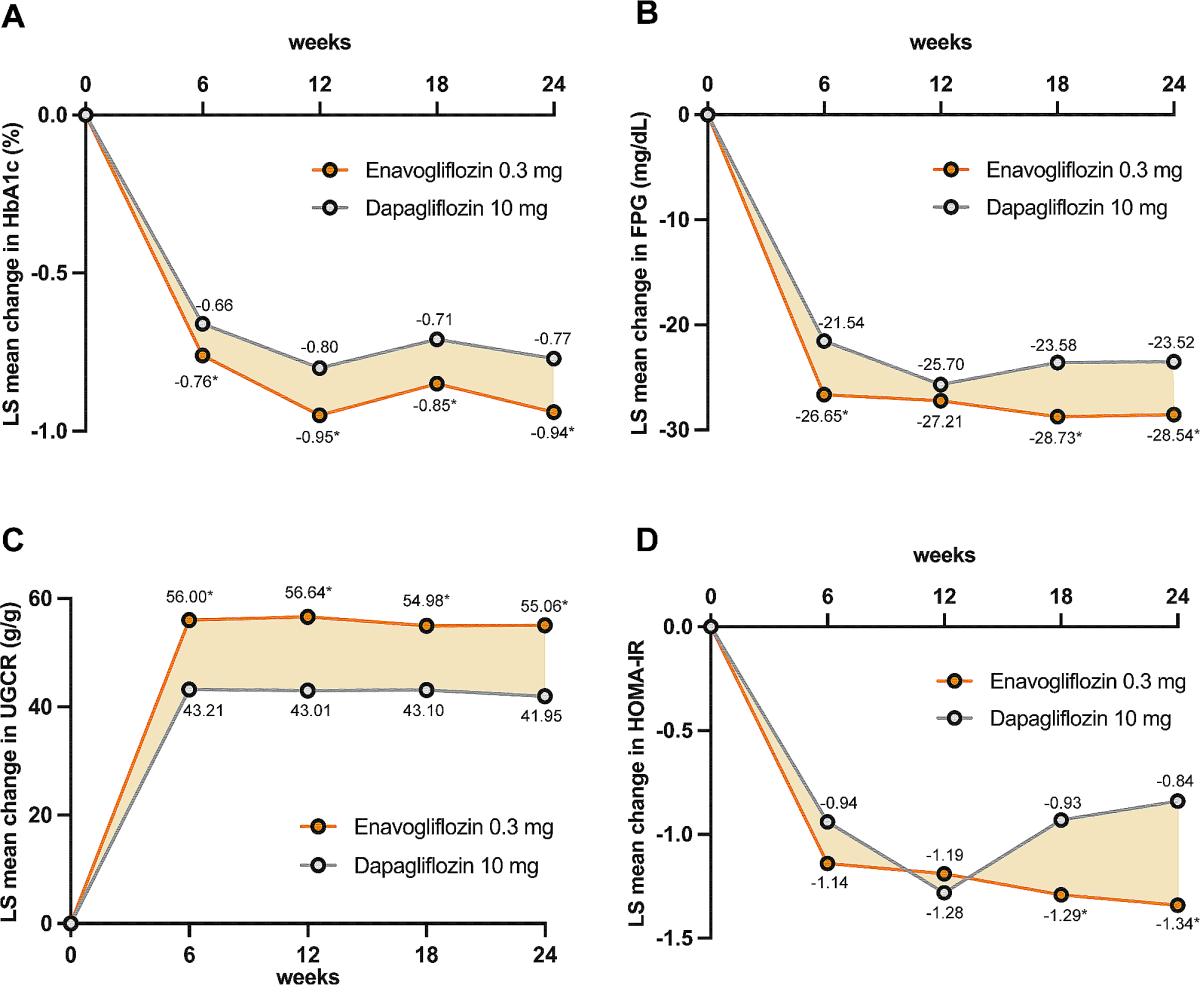
Changes in efficacy parameters over time in patients with mildly reduced kidney function. **A**, Glycated hemoglobin (HbA1c). **B**, Fasting plasma glucose (FPG). **C**, Urine glucose-to-creatinine ratio (UGCR). **D**, Homeostasis model assessment of insulin resistance (HOMA-IR). The asterisks denote a statistically significant difference between the groups (*p < 0.05). LS, least squares

**Fig. 2:**
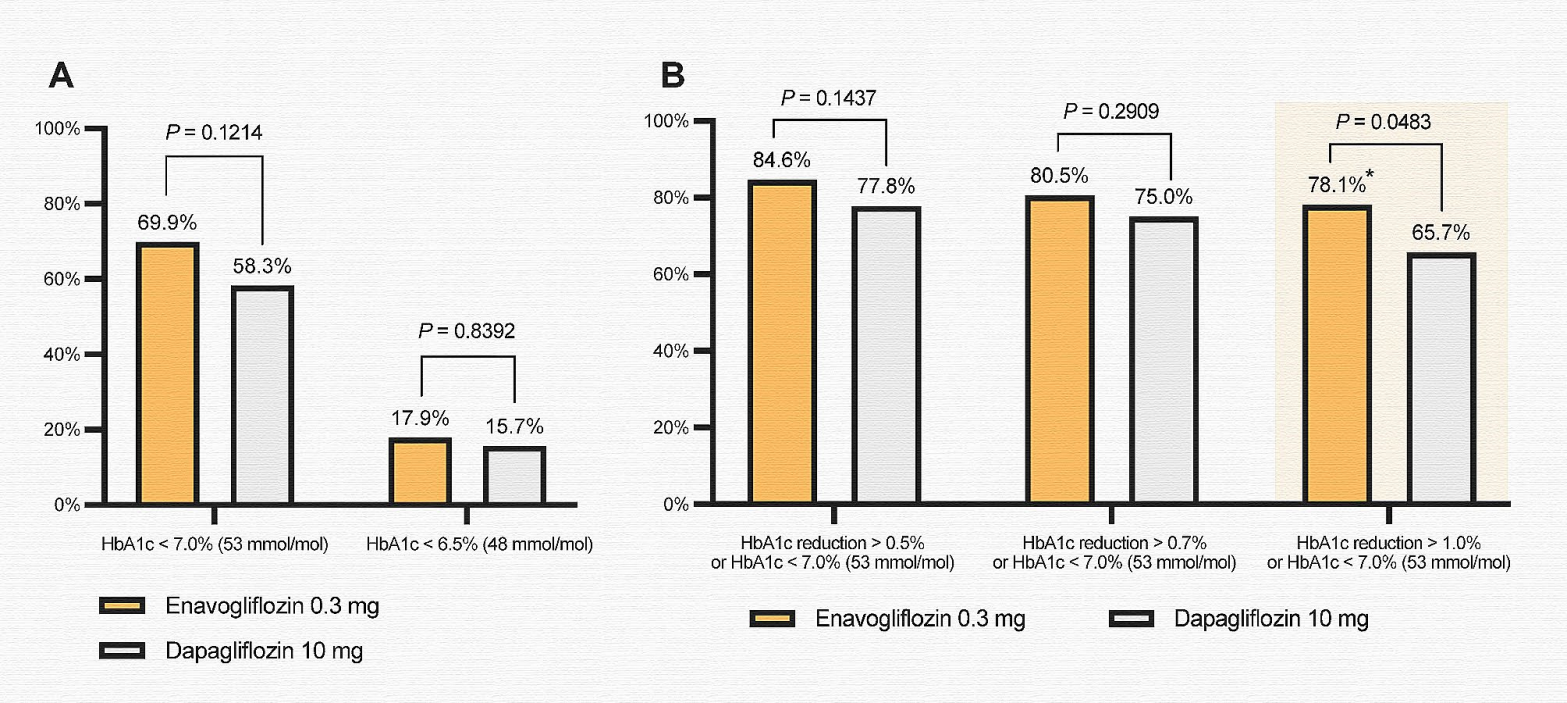
Proportions of patients achieving target HbA1c levels at week 24 (Per-Protocol Set). **A**, Proportions of patients achieving HbA1c less than 7.0% or 6.5% at week 24. **B**, Proportions of patients achieving therapeutic glycemic response at week 24. The asterisks denote a statistically significant difference between the groups (*p < 0.05)

The key efficacy results are presented in Table 3. These results were analyzed separately for the normal and mildly reduced eGFR groups. In the normal eGFR group, at week 24, no significant difference in the HbA1c and FPG levels was found between the two groups (HbA1c: −0.88% [-9.62 mmol/mol] in the enavogliflozin group vs. −0.97% [-10.65 mmol/mol] in the dapagliflozin group, *P* = 0.2063; FPG: −32.01 mg/dL in the enavogliflozin group vs. −30.18 mg/dL in the dapagliflozin group, *P* = 0.4382). Within the mildly reduced eGFR group, the enavogliflozin group showed a significant reduction in the HbA1c and FPG levels compared with the dapagliflozin 10 mg group in week 6, and this effect persisted up to week 24 (HbA1c: −0.94% [-10.26 mmol/mol] in the enavogliflozin group vs. −0.77% [-8.42 mmol/mol] in the dapagliflozin group, *P* = 0.0196; FPG: −28.54 mg/dL in the enavogliflozin group vs. −23.52 mg/dL in the dapagliflozin group, *P* = 0.0371) (Fig. 1). The enavogliflozin 0.3 mg group had a higher proportion of patients with HbA1c levels below 7.0% (53 mmol/mol) (69.9% vs. 58.3%) and below 6.5% (48 mmol/mol) (17.9% vs. 15.7%), and a significantly higher proportion of patients with HbA1c levels below 7.0% (53 mmol/mol) or with a reduction greater than 1.0% (78.1% vs. 65.7%) than the dapagliflozin group at week 24 (Fig. 2). In the subgroup analysis categorized by the combination with or without DPP-4i, baseline age (≥ 65 vs. < 65), BMI (≥ 25 kg/m^2^ vs. < 25 kg/m^2^), HbA1c (≥ 8% [64 mmol/mol] vs. < 8%), and albuminuria (normoalbuminuric vs. microalbuminuria & macroalbuminuria), the glucose-lowering effect of enavogliflozin was more pronounced when used in combination with DPP-4i, in individuals with uncontrolled diabetes (≥ 8% [64 mmol/mol]) and young patients (< 65 years) (Supplemental Figs. 1, 2). Enavogliflozin demonstrated superior blood glucose-lowering efficacy regardless of renal function (HbA1c; −0.84% [-9.16 mmol/mol] in the normal eGFR group vs. −0.96% [-10.50 mmol/mol] in the mildly reduced eGFR group, *P* = 0.0744). However, dapagliflozin treatment resulted in a significant decrease in the blood glucose-lowering efficacy when the renal function was reduced (HbA1c; −0.95% [-10.42 mmol/mol] in the normal eGFR group vs. −0.80% [-8.74 mmol/mol] in the mildly reduced eGFR group, *P* = 0.0488) (Supplemental Table 1).

**Table 3:** Change in the levels of major efficacy parameters from baseline

	Normal eGFR (eGFR ≥ 90 mL/min/1.73m^2^)		Mildly reduced eGFR (60 ≤ eGFR < 90 mL/min/1.73m^2^)	
	Enavogliflozin 0.3 mg (*n* = 91)	Dapagliflozin 10 mg (*n* = 105)	Enavogliflozin 0.3 mg (*n* = 123)	Dapagliflozin 10 mg (*n* = 108)
**HbA1c, %**				
Baseline	7.81 (0.83)	7.78 (0.82)	7.74 (0.77)	7.75 (0.75)
Week 24	7.00 (0.59)	6.88 (0.67)	6.85 (0.55)	7.02 (0.67)
Change from baseline at week 24				
LS mean (SE)	-0.88 (0.06)	-0.97 (0.06)	-0.94 (0.05)	-0.77 (0.06)
LS mean difference [95% Cl], *p*-value^¶^	0.09 [-0.05, 0.24], *p* = 0.2063		-0.17 [-0.31, -0.03], *p* = 0.0196	
**HbA1c, mmol/mol**				
Baseline	61.90 (9.06)	61.55 (8.91)	61.12 (8.39)	61.20 (8.18)
Week 24	53.02 (6.41)	51.75 (7.34)	51.33 (6.01)	53.17 (7.36)
Change from baseline at week 24				
LS mean (SE)	-9.62 (0.69)	-10.65 (0.62)	-10.26 (0.60)	-8.42 (0.63)
LS mean difference [95% Cl], *p*-value^¶^	1.03 [-0.57, 2.63], *p* = 0.2063		-1.84 [-3.39, -0.30], *p* = 0.0196	
**FPG, mg/dL**				
Baseline	146.53 (29.00)	146.62 (30.70)	138.67 (25.87)	141.94 (31.02)
Week 24	114.75 (18.28)	116.39 (18.93)	112.19 (16.71)	118.15 (21.93)
Change from baseline at week 24				
LS mean (SE)	-32.01 (1.97)	-30.18 (1.78)	-28.54 (1.78)	-23.52 (1.93)
LS mean difference [95% Cl], *p*-value^¶^	-1.83 [-6.48, 2.82], *p* = 0.4382		-5.03 [-9.75, -0.30], *p* = 0.0371	
**Body weight, kg**				
Baseline	68.93 (11.94)	70.24 (12.58)	70.23 (11.42)	67.03 (10.51)
Week 24	65.69 (11.50)	67.00 (12.62)	66.72 (11.10)	63.95 (10.00)
Change from baseline at week 24				
LS mean (SE)	-3.25 (0.27)	-3.21 (0.24)	-3.57 (0.30)	-3.39 (0.32)
LS mean difference [95% Cl], *p*-value^¶^	-0.04 [-0.67, 0.59], *p* = 0.9033		-0.18 [-0.96, 0.61], *p* = 0.6587	
**UGCR, g/g**				
Baseline	1.65 (8.88)	2.65 (10.11)	0.66 (3.44)	0.70 (2.92)
Week 24	67.75 (22.21)	47.18 (25.01)	54.78 (21.92)	41.79 (17.37)
Change from baseline at week 24				
LS mean (SE)	64.23 (2.75)^†^	43.79 (2.48)^‡^	55.06 (1.96)	41.95 (2.11)
LS mean difference [95% Cl], *p*-value^¶^	20.44 [13.92, 26.97], *p* < 0.0001		13.11 [7.91, 18.31], *p* < 0.0001	
**HOMA-β**				
Baseline	39.04 (25.37)	43.72 (28.16)	47.79 (34.97)	40.61 (24.55)
Week 24	50.53 (41.70)	54.47 (39.95)	47.03 (65.99)	49.40 (29.01)
Change from baseline at week 24				
LS mean (SE)	14.98 (3.95)	13.96 (3.54)	1.15 (4.62)	9.52 (4.97)
LS mean difference [95% Cl], *p*-value^¶^	1.01 [-8.33, 10.36], *p* = 0.8311		-8.37 [-20.67, 3.93], *p* = 0.1812	
**HOMA-IR**				
Baseline	3.09 (2.18)	3.57 (2.42)	3.19 (3.03)	3.00 (1.92)
Week 24	1.85 (1.21)	2.08 (1.42)	1.77 (1.00)	2.21 (1.72)
Change from baseline at week 24				
LS mean (SE)	-1.35 (0.12)	-1.30 (0.11)	-1.34 (0.13)	-0.84 (0.14)
LS mean difference [95% Cl], *p*-value^¶^	-0.05 [-0.33, 0.24], *p* = 0.7498		-0.50 [-0.84, -0.16], *p* = 0.0038	
**SBP, mmHg** ^§^				
Baseline	127.11 (10.93)	125.47 (12.07)	129.49 (14.54)	125.34 (13.66)
Week 24	122.41 (11.64)	120.85 (11.07)	123.04 (13.25)	120.56 (12.74)
Change from baseline at week 24				
LS mean (SE)	-3.49 (1.11)	-4.24 (1.00)	-6.55 (0.95)	-6.54 (1.02)
LS mean difference [95% Cl], *p*-value^¶^	0.75 [-1.88, 3.38], *p* = 0.5746		-0.02 [-2.55, 2.52], *p* = 0.9901	
**DBP, mmHg** ^§^				
Baseline	76.95 (8.89)	75.84 (8.25)	76.68 (9.87)	73.65 (10.42)
Week 24	73.47 (9.17)	73.00 (8.13)	71.99 (9.56)	71.39 (9.40)
Change from baseline at week 24				
LS mean (SE)	-3.22 (0.81)	-2.98 (0.72)	-4.68 (0.68)	-3.47 (0.73)
LS mean difference [95% Cl], *p*-value^¶^	-0.24 [-2.14, 1.66], *p* = 0.8019		-1.21 [-3.01, 0.60], *p* = 0.1887	

Data are primarily based on the per-protocol set and presented as mean (standard deviation) unless otherwise specified. ^†^As one subject did not have week 24 value, total 90 patients’ data were used for calculation of change from baseline. ^‡^As one subject did not have baseline value, total 104 patients’ data were used for calculation of change from baseline. ^§^Blood pressure data are based on the modified per-protocol set 2 (Enavogliflozin (*n* = 87), Dapagliflozin (*n* = 102) in Normal eGFR group and Enavogliflozin (*n* = 120), Dapagliflozin (*n* = 106) in Mildly reduced eGFR group)

^¶^Testing for difference between Enavogliflozin and Dapagliflozin (ANCOVA with treatment group as a factor, baseline value and stratification factors as covariates)

DBP: diastolic blood pressure; FPG: fasting plasma glucose; HbA1c: glycated hemoglobin; HOMA-β: homeostasis model assessment of β-cell function; HOMA-IR: homeostasis model assessment of insulin resistance; SBP: systolic blood pressure; UACR: urine albumin-to-creatinine ratio; UGCR; urine glucose-to-creatinine ratio

In the normal eGFR group, the body weight decreased in both the enavogliflozin and dapagliflozin groups and showed no significant difference between the two groups (− 3.25 kg in the enavogliflozin group vs. −3.21 kg in the dapagliflozin group, *P* = 0.9033) (Table 2). No significant differences in the HOMA-β or HOMA-IR, or in the systolic or diastolic blood pressure were observed between the two drug groups. In the mildly reduced eGFR group, the body weight gradually decreased in both the enavogliflozin and dapagliflozin groups and no significant difference between the two groups was observed (− 3.57 kg in the enavogliflozin group vs. −3.39 kg in the dapagliflozin group, *P* = 0.6587). Moreover, the HOMA-IR values improved more with enavogliflozin than with dapagliflozin (mean change: −0.50, *P* = 0.0038). No significant differences in SBP or DBP were observed between the two groups in the mildly reduced eGFR group.

**Fig. 3:**
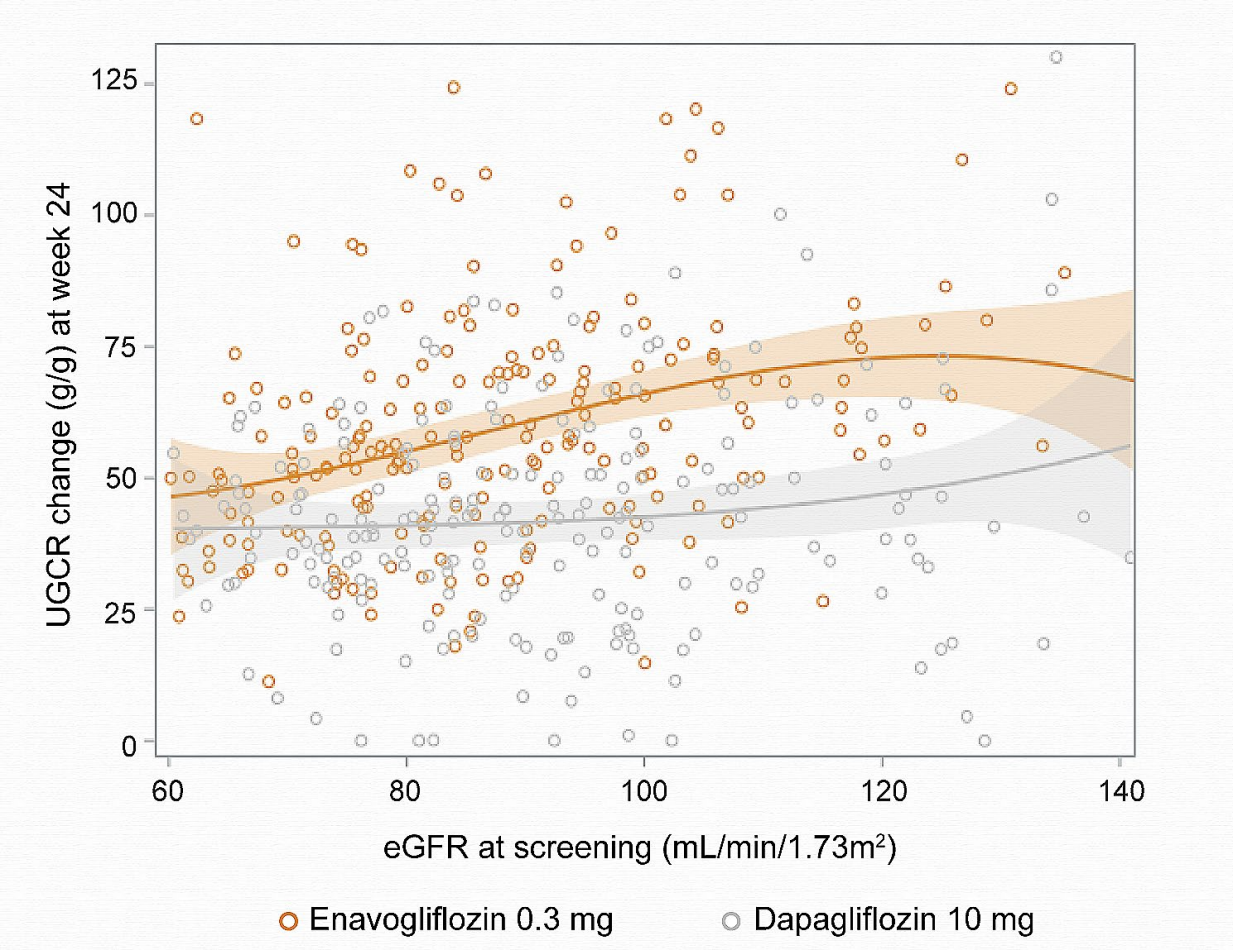
The relationship between eGFR and the change in urine glucose-to-creatinine ratio from baseline. The horizontal line shows the continuous UGCR change from baseline across the range of eGFR and the shaded area around this line represents the 95% CI obtained via Pearson’s correlation analysis

The UGE ratio increased to a significantly higher degree with enavogliflozin than with dapagliflozin. In the normal eGFR group, the mean change in UGCR from the baseline was higher in the enavogliflozin group than in the dapagliflozin group (64.23 g/g in the enavogliflozin group vs. 43.79 g/g in the dapagliflozin group, *P* < 0.0001) (Table 3). In the mildly reduced eGFR group, the mean change in UGCR from the baseline was higher in the enavogliflozin group (55.06 g/g) than in the dapagliflozin group (41.95 g/g). The changes in UGCR at week 24 from baseline are displayed in Fig. 3, in which the UGCR based on the eGFR at screening is plotted. The plot indicates that enavogliflozin resulted in higher UGCR values than dapagliflozin across all eGFR values. Based on the various eGFR ranges, enavogliflozin led to a greater UGE than dapagliflozin (Supplemental Table 2).

### Safety

Supplemental Table 3 presents the adverse events observed in the pooled analysis. The incidence of TEAEs with enavogliflozin and dapagliflozin treatment was 22.55% and 22.98%, respectively, with no significant differences being observed. The incidence of serious TEAEs with enavogliflozin and dapagliflozin treatment was 1.28% and 2.98%, respectively. Among the TEAEs of special interest, two cases of vaginal infection and one case of cystitis were reported in each of the groups, whereas one case each of genital infection, vulvovaginal candidiasis, vulvovaginitis, and pollakiuria occurred in the dapagliflozin group. Additionally, one hypoglycemia case was reported in each of the groups.

## Discussion

The pooled analysis of data from two randomized controlled phase III trials aimed to demonstrate the efficacy and safety of enavogliflozin as an add-on therapy and to compare it to dapagliflozin for patients with type 2 diabetes mellitus classified according to the eGFR. Our results demonstrate that once-daily treatment with enavogliflozin 0.3 mg resulted in significantly greater reductions in the HbA1c and FPG levels than once-daily treatment with dapagliflozin 10 mg as an add-on therapy in patients with a mildly reduced renal function. In addition, enavogliflozin led to a greater increase of UGE than dapagliflozin across all investigated eGFR values, suggesting a possible mechanism that explains the greater blood glucose-lowering effect of enavogliflozin compared to that of dapagliflozin.

Understanding and addressing kidney dysfunction is pivotal in managing and treating diabetic patients. Chronic kidney disease, a prominent microvascular complication of diabetes, is a critical factor contributing to increased mortality and morbidity among individuals with diabetes [10]. The recent research of SGLT-2 inhibitors revealing the lower incidence of chronic kidney disease (CKD) progression have established them as essential medications for individuals with diabetic nephropathy [11, 12]. From this perspective, the emergence of enavogliflozin, a novel and potent SGLT-2 inhibitor, can serve as a new tool for patients with diabetes and chronic kidney diseases.

Marked glucose-lowering efficacy without leading to hypoglycemia is an important advantage of glucose-lowering medications. SGLT-2 inhibitors are an attractive and favorable option since they demonstrate a potent blood glucose-lowering effect with a low risk of hypoglycemia, owing to their insulin-independent mechanism of action. SGLT-2 inhibitors lower the blood glucose level by promoting glucose excretion through the kidneys. As expected, the glucose-lowering efficacy of SGLT-2 inhibitors diminished as the kidney function declined. This is a potential drawback of using SGLT-2 inhibitors in patients with impaired kidney function. However, our study demonstrated that enavogliflozin 0.3 mg showed potent glucose-lowering efficacy compared to that of dapagliflozin 10 mg in patients with a mildly reduced kidney function, with an eGFR of ≥ 60 to < 90 mL/min/1.73 m². An intriguing finding emerged when the blood glucose-lowering efficacy of each medication was analyzed separately based on the renal function. Enavogliflozin showed potent blood glucose-lowering effects regardless of renal function, whereas the blood glucose-lowering effects of dapagliflozin decreased significantly as renal function declined (Supplemental Table 1). Because of the eGFR-dependent glucose-lowering effect of SGLT-2 inhibitors, individuals with chronic kidney disease may benefit more by using a SGLT-2 inhibitor with high post-treatment UGE. In this respect, enavogliflozin may offer greater advantages to these patients.

Our study showed that the glucose-lowering effect of enavogliflozin was more pronounced than that of dapagliflozin, starting as early as 6 weeks after administration. This fast and potent glucose-lowering effect was consistently observed for both the HbA1c and FPG levels. The rapid and marked effectiveness of diabetes medication in the early stages of administration is not only important for achieving glycemic control but also helps reduce the long-term risk of diabetes treatment failure. KJ Kim et al. reported that newly diagnosed diabetes patients who quickly reached the target blood glucose levels (< 3 months) had fewer microvascular complications than those who achieved the target levels later (≥ 6 months) [13]. Furthermore, the group that achieved the target glucose levels early exhibited better long-term glycemic control, indicating improved pancreatic beta cell function. Another study conducted by Laiteerapong et al. reported similar results [14]. They reported that achieving good blood glucose control within the first year after the initial diabetes diagnosis not only reduces the risk of microvascular complications but also lowers the mortality rate. The association between rapid blood glucose control and favorable clinical outcomes is related to pancreatic beta cell function improvements. Quickly achieving the treatment goals for newly diagnosed patients with type 2 diabetes mellitus can also aid pancreatic beta cells in escaping glucose toxicity early on [15]. The rapid and marked blood glucose-lowering effect of enavogliflozin is expected to result in favorable outcomes in future studies.

An interesting finding of our study was that the post-treatment UGE change was significantly higher for enavogliflozin 0.3 mg than for dapagliflozin 10 mg, regardless of the eGFR value. Considering the mechanism of action of SGLT-2 inhibitors, these findings can explain the potent enavogliflozin glucose-lowering effect. The phase 1 study of enavogliflozin 0.3 mg showed that it led to a greater post-treatment UGE than dapagliflozin 10 mg in healthy subjects [6]. The relationship between the change in UGE and the glucose-lowering efficacy of SGLT-2 inhibitors in patients with type 2 diabetes mellitus has been researched; however, the conclusions drawn are inconsistent. In a study involving 20 Japanese participants with type 2 diabetes mellitus, dapagliflozin was more effective in reducing blood glucose levels in younger patients than in older individuals [16]. This was demonstrated by a greater degree of UGE changes being observed in the younger age group. Another study by Kim et al. reported that a morning UGCR increase was correlated with a HbA1c level reduction after administering dapagliflozin 10 mg or ipragliflozin 50 mg [17]. Interestingly, the observed correlation could not be consistently applied in all cases, as in some cases, the morning UGE did not increase but significant blood glucose-lowering effects were observed. A similar outcome was observed in a study that compared canagliflozin 300 mg and dapagliflozin 10 mg. Treatment with canagliflozin 300 mg resulted in a higher UGE rate than with dapagliflozin 10 mg [18]. However, no significant difference between the glucose-lowering effects of the two drugs was observed [19]. Our results also showed, especially in the normal eGFR group, that no significant difference in the HbA1c or FPG levels was observed between the two groups (HbA1c: −0.88% [-9.62 mmol/mol] in the enavogliflozin group vs. −0.97% [-10.65 mmol/mol] in the dapagliflozin group; FPG: −32.01 mg/dL in the enavogliflozin group vs. −30.18 mg/dL in the dapagliflozin group). The inconsistency in the blood glucose-lowering effects noted in these studies suggests that, while UGE is an important mechanism for blood glucose level reduction by SGLT-2 inhibitors, other factors could also affect the blood glucose-lowering effects. Particularly, in states of very high blood glucose levels, the natural overflow glycosuria increases. Therefore, considering the impact of natural overflow glucosuria within UGE in patients with high blood glucose levels is essential [17]. Additionally, an increase in glucagon concentration, which can suppress the blood glucose-lowering effects of SGLT-2 inhibitors, should also be considered. The non-insulin-dependent mechanism of SGLT-2 increases endogenous glucose production by increasing the glucagon levels in the body, which is a potential confounder when assessing the relationship between UGE and the blood glucose-lowering effect [20]. These hypotheses could be explained by mechanisms accounting for the lack of significant differences among patients with an eGFR ≥ 90 between the enavogliflozin and dapagliflozin groups observed here, despite the fact that enavogliflozin induced high UGE. In addition, in our study, the efficacy of enavogliflozin was more pronounced when combined with DPP-4 inhibitors. This result can be explained by the suppression of SGLT-2 inhibitor-induced glucagon secretion by DPP-4 inhibitors. If glucagon is suppressed, the blood glucose-lowering effect of SGLT-2 inhibitors would be more closely related to UGE. Therefore, the enhanced effect of enavogliflozin when combined with DPP-4 inhibitors, as compared to dapagliflozin, can be explained by the mechanism in this context. Further studies are needed to explore the relationship between UGE and its glucose-lowering effect in individuals with a normal eGFR.

Notably, we showed that the HOMA-IR, represented by insulin resistance, improved in the enavogliflozin group. In particular, in patients with a mildly reduced eGFR, enavogliflozin significantly improved the HOMA-IR compared to dapagliflozin. Similar results have been reported in other phase III studies of enavogliflozin that aimed to assess the efficacy of enavogliflozin monotherapy in drug-naïve patients [21]. The HOMA-IR improvement could be explained by the mechanism underlying the potent blood glucose-lowering effect of enavogliflozin. Insulin resistance is a fundamental type 2 diabetes mellitus mechanism; therefore, the HOMA-IR improvement can be explained as a mechanism that improves the blood glucose levels in patients with type 2 diabetes mellitus. SGLT-2 inhibitors significantly enhance insulin sensitivity and improve glucose toxicity in pancreatic beta cells through their anti-inflammatory effects [22–24]. However, the specific mechanism by which enavogliflozin does this compared to other SGLT-2 inhibitors is still unclear; therefore, further research is needed.

### Study limitations

Our study has some key limitations. First, although it is a pooled analysis, its relatively small sample size and short-term nature limited the ability to conclusively determine the efficacy difference between the two drugs. Second, we did not include patients with an eGFR below 60 mL/min/1.73 m²; these are patients with CKD with a more advanced kidney impairment. Third, our results were mainly statistically significant in patients with an eGFR < 90 mL/min/1.73 m², which raises the possibility of having been discovered by chance. To address this limitation and demonstrate that this is unlikely, we performed subgroup analyses (shown in the supplementary data). Finally, the data used here were obtained from participants involved in tightly controlled clinical trials, and their demographic and clinical characteristics may not fully represent those of the broader population of patients with type 2 diabetes mellitus receiving SGLT-2 inhibitors in clinical practice. Real-world experiences may differ from the findings of controlled trials and safety results should be interpreted accordingly.

## Conclusions

This pooled analysis demonstrates that enavogliflozin as an add-on therapy results in significant HbA1c and FPG level reductions compared with dapagliflozin in patients with mildly reduced renal function. Additionally, enavogliflozin showed potent blood glucose-lowering effects regardless of the renal function, whereas dapagliflozin exhibited a significant decrease in its blood glucose-lowering effects when the renal function declined. These results can be explained by the mechanism by which enavogliflozin exhibits a greater UGE increase across all eGFR values. The development of powerful SGLT-2 inhibitor provides a valuable treatment option for patients with poor glucose control and decreased renal function.

### Electronic supplementary material

Below is the link to the electronic supplementary material.

Supplementary Material 1

## Data Availability

The dataset supporting the findings of this study is available from Cheol-Young Park, but the availability of this data is limited. This data is used under license for current research and is not publicly available. However, the authors can provide the data upon reasonable request and with the permission of Cheol-Young Park.

## Abbreviations

DPP-4: Dipeptidyl peptidase-4
eGFR: Estimated glomerular filtration rate
FPG: Fasting plasma glucose
HbA1c: Hemoglobin A1c
HOMA-B: Homeostasis model assessment of beta-cell function
HOMA-IR: Homeostasis model assessment of insulin resistance
SGLT-2: Sodium-glucose cotransporter-2
UGCR: Urine glucose-to-creatinine ratio
UGE: Urinary glucose excretion
