# Supplementary material for: Efficacy and safety of enavogliflozin vs. dapagliflozin as add-on therapy in patients with type 2 diabetes mellitus based on renal function: a pooled analysis of two randomized controlled trials
Source: Cardiovasc Diabetol. 2024 Feb 15;23:71. doi: 10.1186/s12933-024-02155-9 (PMC10870449; doi:10.1186/s12933-024-02155-9)
Supplement: Supplementary file 1 — Supplementary Material 1 [file 12933_2024_2155_MOESM1_ESM.docx]

**Supplementary material - Efficacy and safety of enavogliflozin vs. dapagliflozin as add-on therapy in patients with type 2 diabetes mellitus based on renal function: A pooled analysis of two randomized controlled trials**

**Running title**: Efficacy of enavogliflozin for type 2 diabetes

**[author names]**

Young Sang Lyu^1^, Sangmo Hong^2^, Si Eun Lee^3^, Bo Young Cho^3^, Cheol-Young Park^4^

**[author affiliations]**

^1^Division of Endocrinology and Metabolism, Department of Internal Medicine, Chosun University Hospital, Gwangju, Republic of Korea.

^2^Division of Endocrinology, Department of Internal Medicine, Hanyang University Guri Hospital, 153 Gyeongchun-ro, Guri 11923, Korea.

^3^Daewoong Pharmaceutical Co., Ltd., Seoul, Republic of Korea.

^4^Department of Internal Medicine, Kangbuk Samsung Hospital, Sungkyunkwan University School of Medicine, Seoul, Republic of Korea.

**Corresponding Author:**

Cheol-Young Park

Division of Endocrinology and Metabolism, Department of Internal Medicine, Kangbuk Samsung Hospital, Sungkyunkwan University School of Medicine, 29 Saemunan-ro,

Jongno-gu, Seoul 03181, Korea

E-mail: cydoctor@chol.com

**Supplementary Tables**

**Supplemental Table 1.** Changed from baseline in glucose lowering efficacy based on renal function

|  | Enavogliflozin 0.3 mg  (n=214) | | Dapagliflozin 10 mg  (n=213) | |
| --- | --- | --- | --- | --- |
|  | **Normal eGFR**  **(n=91)** | **Mildly reduced eGFR**  **(n=123)** | **Normal eGFR**  **(n=105)** | **Mildly reduced eGFR**  **(n=108)** |
| HbA1c, % |  |  |  |  |
| Baseline | 7.81 (0.83) | 7.74 (0.77) | 7.78 (0.82) | 7.75 (0.75) |
| Week 24 | 7.00 (0.59) | 6.85 (0.55) | 6.88 (0.67) | 7.02 (0.67) |
| Change from baseline at week 24 |  |  |  |  |
| LS mean (SE) | -0.84 (0.06) | -0.96 (0.05) | -0.95 (0.06) | -0.80 (0.06) |
| LS mean difference [95% Cl], *p*-value^⁋^ | 0.12 [-0.01, 0.26], *p*=0.0744 | | -0.15 [-0.31, 0.00], *p*=0.0488 | |
| HbA1c, mmol/mol |  |  |  |  |
| Baseline | 61.90 (9.06) | 61.12 (8.39) | 61.55 (8.91) | 61.20 (8.18) |
| Week 24 | 53.02 (6.41) | 51.33 (6.01) | 51.75 (7.34) | 53.17 (7.36) |
| Change from baseline at week 24 |  |  |  |  |
| LS mean (SE) | -9.16 (0.66) | -10.50 (0.55) | -10.42 (0.67) | -8.74 (0.66) |
| LS mean difference [95% Cl], *p*-value^⁋^ | 1.34 [-0.13, 2.82], *p*=0.0744 | | -1.68 [-3.35, -0.01], *p*=0.0488 | |
| FPG, mg/dL |  |  |  |  |
| Baseline | 146.53 (29.00) | 138.67 (25.87) | 146.62 (30.70) | 141.94 (31.02) |
| Week 24 | 114.75 (18.28) | 112.19 (16.71) | 116.39 (18.93) | 118.15 (21.93) |
| Change from baseline at week 24 |  |  |  |  |
| LS mean (SE) | -30.13 (1.86) | -29.85 (1.53) | -28.45 (2.06) | -25.81 (2.04) |
| LS mean difference [95% Cl], *p*-value^⁋^ | -0.28 [-4.54, 3.98], *p*=0.8969 | | -2.65 [-7.85, 2.56], *p*=0.3177 | |

Data are primarily based on the per-protocol set and presented as mean (standard deviation).

^⁋^Testing for difference between Normal eGFR group and Mild reduced eGFR group (ANCOVA with eGFR group as a factor, baseline value and stratification factors as covariates).

FPG, fasting plasma glucose; HbA1c, glycated hemoglobin.

**Supplemental Table 2.** UGCR Change from baseline at week 24 according to eGFR levels

| eGFR level | Enavogliflozin 0.3 mg (n=213) | | Dapagliflozin 10 mg (n=212) | |
| --- | --- | --- | --- | --- |
|  | n | Mean (SD) | n | Mean (SD) |
| 60 ≤ eGFR ‹ 75 | 46 | 47.21 (18.36) | 39 | 40.13 (15.00) |
| 75 ≤ eGFR ‹ 90 ^†^ | 77 | 58.25 (22.73) | 69 | 41.63 (18.98) |
| 90 ≤ eGFR ‹ 105 ^†^ | 55 | 63.68 (22.24) | 57 | 40.75 (22.76) |
| 105 ≤ eGFR ‹ 120 | 23 | 66.39 (20.74) | 24 | 50.98 (23.92) |
| 120 ≤ eGFR | 12 | 76.52 (25.35) | 23 | 45.56 (31.06) |

Data are primarily based on the per-protocol set and presented as mean (standard deviation).

^†^Testing for difference between Enavogliflozin and Dapagliflozin (ANCOVA with treatment group as a factor, baseline value and stratification factors as covariates). Statistical significance was assessed at a threshold of less than 0.01 to reduce false positives to adjustment for multiple comparisons.

eGFR, estimated glomerular filtration rate; N, number.

**Supplemental Table 3**. Summary of adverse events

|  | Enavogliflozin 0.3 mg  (n=235) | Dapagliflozin 10 mg  (n=235) |
| --- | --- | --- |
| TEAEs, n (%) | 53 (22.55) [75] | 54 (22.98) [80] |
| Serious TEAEs, n (%) | 3 (1.28) [3] | 7 (2.98) [8] |
| TEAEs leading to drug discontinuation | 0 | 1 (0.43) [1] |
| TEAEs leading to death | 0 | 0 |
| TEAEs of special interest | 4 (1.70) [4] | 8 (3.40) [8] |
| Vaginal infection | 2 (0.85) [2] | 2 (0.85) [2] |
| Cystitis | 1 (0.43) [1] | 1 (0.43) [1] |
| Genital infection | 0 | 1 (0.43) [1] |
| Vulvovaginal candidiasis | 0 | 1 (0.43) [1] |
| Vulvovaginitis | 0 | 1 (0.43) [1] |
| Hypoglycaemia | 1 (0.43) [1] | 1 (0.43) [1] |
| Pollakiuria | 0 | 1 (0.43) [1] |
| ADRs, n (%) | 7 (2.98) [7] | 14 (5.96) [14] |
| Vaginal infection | 2 (0.85) [2] | 2 (0.85) [2] |
| Cystitis | 1 (0.43) [1] | 1 (0.43) [1] |
| Genital infection | 0 | 1 (0.43) [1] |
| Vulvovaginal candidiasis | 0 | 1 (0.43) [1] |
| Vulvovaginitis | 0 | 1 (0.43) [1] |
| Pruritus | 1 (0.43) [1] | 2 (0.85) [2] |
| Dyspepsia | 0 | 2 (0.85) [2] |
| Lethargy | 1 (0.43) [1] | 0 |
| Somnolence | 1 (0.43) [1] | 0 |
| Ocular discomfort | 0 | 1 (0.43) [1] |
| Blood pressure decreased | 1 (0.43) [1] | 0 |
| Hypoglycaemia | 0 | 1 (0.43) [1] |
| Pollakiuria | 0 | 1 (0.43) [1] |
| Vulvovaginal pruritus | 0 | 1 (0.43) [1] |

Data are number of subjects (%) [number of events]. Data were analyzed using the safety set.

ADR, adverse drug reaction; TEAE, treatment-emergent adverse event.

**Supplementary Figure**

**
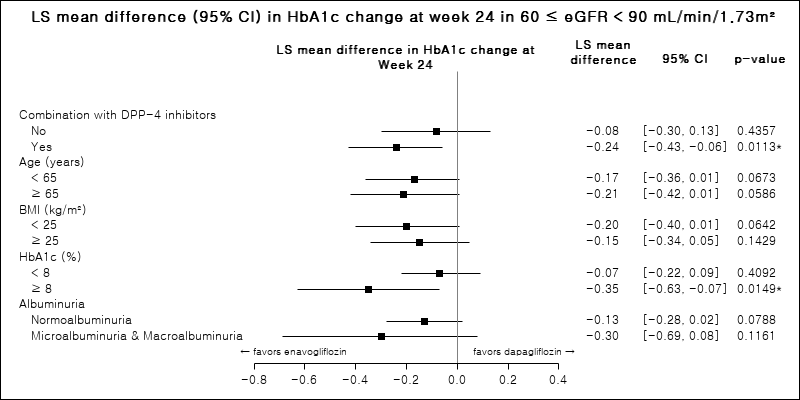
**

**Supplemental Figure 1. Least-square mean difference (95% confidence interval) in the HbA1c level change at week 24 in patients with a mildly reduced kidney function, with an eGFR of ≥ 60 to < 90 mL/min/1.73 m² (Per-Protocol Set). BMI, body mass index; CI, confidence interval; DPP-4, dipeptidyl peptidase-4 inhibitors-4; eGFR, estimated glomerular filtration rate; LS, least square; OHA, oral antihyperglycemic agent.**

**
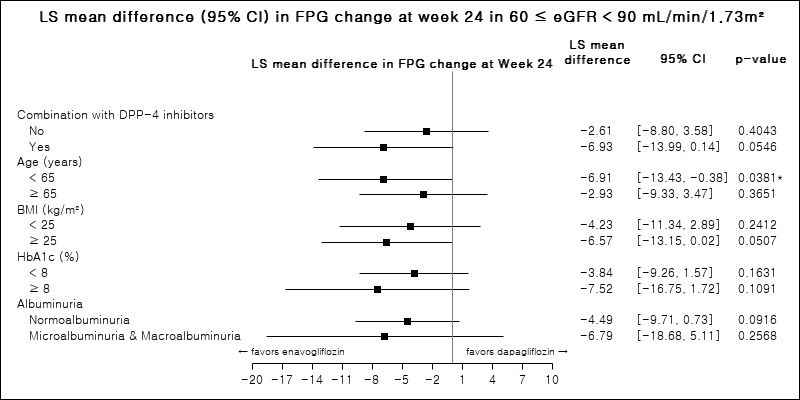
**

**Supplemental Figure 2. Least-square mean difference (95% confidence interval) in the fasting plasma glucose level change at week 24 in patients with a mildly reduced kidney function, with an eGFR of ≥ 60 to < 90 mL/min/1.73 m² (Per-Protocol Set). BMI, body mass index; CI, confidence interval; DPP-4, dipeptidyl peptidase-4 inhibitors-4; eGFR, estimated glomerular filtration rate; FPG, fasting plasma glucose; HbA1c, LS, least square; OHA, oral antihyperglycemic agent.**
